# Causal relationships between antibody-mediated immune responses and acute pancreatitis: Evidence from a genetic study

**DOI:** 10.1097/MD.0000000000045223

**Published:** 2025-10-17

**Authors:** Minjian Xie, Liqun Li, Sheng Xie, Xiaoyun Lei, Chengning Yang, Jianfeng Li, Lijian Liu

**Affiliations:** a Faculty of Graduate Studies, Guangxi University of Chinese Medicine, Nanning, Guangxi, China; b Department of Gastroenterology, The First Affiliated Hospital of Guangxi University of Chinese Medicine, Nanning, Guangxi, China.

**Keywords:** acute pancreatitis, antibody-mediated immune responses, inflammatory cytokines, mediation analysis, two-sample bidirectional Mendelian randomization study

## Abstract

Certain specific antibody-mediated immune responses may be associated with acute pancreatitis (AP), but their causal relationship remains uncertain. Therefore, we used bidirectional two-sample Mendelian randomization (MR) to investigate their causal link and potential mediation by inflammatory cytokines.

Data for this study were sourced from a large-scale Genome Wide Association Study (GWAS) communal data pool. To explore the causality between antibody-mediated immune responses and AP, we performed two-sample bidirectional MR analyses using 5 approaches: inverse-variance weighted (IVW), MR-Egger, weighted mode, weighted median, and simple mode. We also studied the potential mediating effect of 91 circulating inflammatory cytokines using a two-step MR method. Additionally, sensitivity analyses were conducted using MR-Egger intercept test and Cochran *Q* test to ensure the robustness of the outcomes. The results of forward MR analysis showed that anti-Epstein-Barr virus (anti-EBV) IgG seropositivity [OR = 0.941; 95% CI, 0.893–0.992; *P* = .023] and human herpes virus (HHV) 6 IE1A antibody levels [OR = 0.894; 95% CI, 0.816–0.981; *P* = .017] significantly reduced the risk of AP. The results of the reverse MR analysis revealed a negative correlation between AP and anti-EBV IgG seropositivity [OR = 0.775; 95% CI, 0.605–0.992; *P* = .043]. Furthermore, none of the 91 circulating inflammatory cytokines could mediate the causal relationship between HHV-6 IE1A antibody levels and the risk of AP. The results of sensitivity analysis confirmed the robustness of these causalities. The current study suggests that HHV-6 IE1A antibody levels are a protective factor against AP, and there is a bidirectional causality between AP and anti-EBV IgG seropositivity. In addition, the mediation analysis results showed that the 91 circulating inflammatory cytokines could not serve as mediators between the 46 antibody-mediated immune responses and AP.

## 1. Introduction

Acute pancreatitis (AP) is a common digestive system disease that causes acute inflammation and acinar cell damage in the pancreas after the deviant activation of pancreatic enzymes.^[1]^ The main symptom is sudden, persistent upper abdominal pain, which may be accompanied by nausea, vomiting, abdominal distension, and fever. Epidemiological investigations have demonstrated that the global incidence rate of AP is approximately 34.8 cases per 100,000 person-years, with the morbidity in Eastern European countries reaching 79.6 cases per 100,000 person-years – an 11% increase compared to 10 years ago.^[2]^ In China, the prevalence of AP among hospitalized patients is about 26.8%, the second-highest worldwide, with a mortality rate ranked third globally.^[2]^ Clinically, while most AP patients present with mild AP, around 20% to 30% may progress to severe acute pancreatitis (SAP).^[3]^ SAP is one of the most serious types of AP. It often involves necrosis and infection of the pancreas or peripancreatic tissues and is associated with organ failure lasting more than 48 hours, posing a serious threat to human life and health.^[4]^

Although progress has been made in understanding the pathogenesis, diagnosis, and treatment of AP in recent years, this has improved the prognosis for some patients.^[5]^ However, the incidence of AP continues to rise annually, with a mortality rate remaining high (5%–10%), and up to 40% for SAP patients. This also increases the prevalence of chronic pancreatitis, diabetes, and other diseases.^[4–6]^ These findings indicate that AP poses a significant threat to human health and life. Exploring the etiology of AP and clarifying its pathogenesis are crucial for reducing its incidence, improving treatment success rates, and alleviating the economic burden on patients.

Gallstone disease and alcohol consumption are the most frequent causes of AP, but about 10% of cases are due to infectious causes, such as viral, bacterial, and parasitic infections.^[7]^ A clinical study found that 12.5% of AP patients had bacterial infections and 65.3% had viral infections, with higher mortality in those with viral infections, indicating that viral infection is a major infectious cause of AP.^[8]^ For instance, adults infected with EB virus are more likely to develop AP^[9]^; seropositivity for Coxsackievirus and echovirus was detected in 19.8% of AP patients.^[10]^ Clinical reports identified viral infections such as herpes simplex virus (HSV), Coxsackievirus, mumps virus, Cytomegalovirus, human immunodeficiency virus (HIV), and Epstein–Barr virus (EBV) as rare causes of AP.^[11]^ These findings suggest that viral infections play a significant role in the onset and progression of AP. Besides viral infections, intestinal bacterial infections may also greatly affect the mortality rate of severe pancreatitis.^[12]^ However, these findings are often influenced by confounding factors, weakening the evidence. Therefore, clarifying the etiology of AP is crucial for predicting its progression, enhancing prevention and treatment strategies, and thereby improving prognosis.

The core pathological mechanism of microbial infection involves a dysregulated immune response of the host to pathogen infection. Antibodies refer to the immunoglobulins produced by B lymphocytes under antigen stimulation, which can specifically bind with the corresponding antigen.^[13]^ Antibodies play a crucial role in maintaining internal homeostasis and defending mammals against pathogens.^[13]^ The immune response is the body’s defensive reaction to pathogens or foreign substances through the immune system, essentially an antigen-antibody reaction. The specific binding of antigens to antibodies or cell surface targets is a critical step in initiating immune regulatory responses and activating the immune system against pathogens.^[14]^ Antibody seropositivity testing can provide key clues to immune exposure and infectious agents.^[15]^ Therefore, measuring the antibody immune response to infection is a commonly used method for investigating the correlation between infectious pathogens and AP.

A cohort study reported that 23.2% of AP patients had significantly elevated titers of Coxsackievirus B or mumps virus antibodies.^[16]^ Microbial infections or autoantigens can activate the immune system of the body, triggering a potent inflammatory cascade response.^[17]^ Research has shown that inflammatory mediators are crucial for the occurrence and development of AP, among which the release of pro-inflammatory cytokines such as TNF-α, IL-9A and IL-8 can lead to an amplified inflammatory cascade effect.^[18–20]^

These findings highlight that antibody-mediated immune responses are crucial in the key pathological processes of AP, with inflammatory cytokines potentially playing a vital role. However, because these findings were derived from clinical observational studies, they are susceptible to various confounding factors, resulting in relatively weak evidence. As a result, the causality between AP and antibody-mediated immune responses, and the mediating role of inflammatory cytokines remain unclear. Thus, designing a precise and comprehensive study to assess the causality between AP risk and antibody-mediated immune responses is crucial for clinical research and improving AP prevention strategies.

Mendelian randomization (MR) is an emerging research method that infers the causalities between exposures and outcomes by using genetic variants strongly associated with exposures as instrumental variables (IVs).^[21]^ Compared to observational studies, MR analysis is based on the natural random allocation of genetic variants according to Mendel laws of inheritance, making it less prone to confounding factors and more credible. MR studies, therefore, have a unique and reliable advantage in investigating causality and have been widely applied to infer causalities between risk factors and disease outcomes. Building on previous research, we propose the following scientific questions: Is there a causality between predicted antibody-mediated immune responses and AP? If such a relationship exists, do inflammatory cytokines act as mediators between them? As far as we know, no previous research has comprehensively explored the causal link between antibody-mediated immune responses and AP by employing a two-sample bidirectional MR approach or investigated the mediating role of inflammatory cytokines. Therefore, this study employed a bidirectional two-sample MR method to explore the potential causality between antibody-mediated immune responses and AP. In addition, the current study applied a two-step MR analysis to evaluate whether inflammatory cytokines function as mediators. This study provides theoretical support for understanding the pathogenesis, early screening, and prevention of AP, and offers a reference for clinical research and the management of AP from the perspective of antibody-mediated immune responses.

## 2. Materials and methods

### 2.1. Study design

We performed a two-sample bidirectional MR analysis. In the forward MR analysis, antibody immune response factors were treated as exposures and AP as the outcome. In the reverse MR analysis, AP was treated as the exposure and antibody immune response factors as the outcomes to clarify whether there is a causality between them. First, we performed a two-sample bidirectional MR analysis on 46 antibody immune response factors and AP to assess the potential causality between them. Subsequently, we used a two-step MR analysis, utilizing 91 circulating inflammatory cytokines as intermediaries, to assess whether they play an intermediary effect in the causality between antibody-mediated immune response factors and AP.

In MR analysis, genetic variants are used as IVs, which are required to meet 3 key hypotheses^[22]^: it is necessary for the genetic variants to be closely related to the exposure; the genetic variants must be completely independent from other confounding factors between antibody-mediated immune responses and AP; and genetic variants should not directly influence the outcome through any pathway other than the exposure variable.

**Figure 1.**
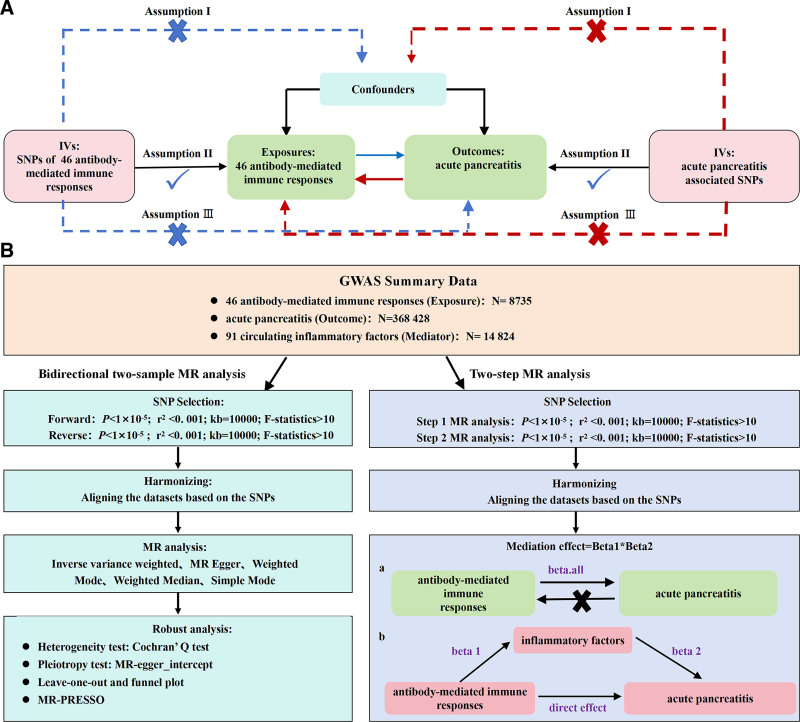
(A) Schematic diagram of the principles of MR. (B) Flowchart of the bidirectional two-sample MR analysis. MR = Mendelian randomization.

All research data are publicly available and have received approval from the related Institutional Review Boards (IRBs). The reporting of our results followed the guidelines outlined in the STROBE-MR for MR studies.^[23]^ The design and assumptions of this research are shown in Figure 1A.

### 2.2. Data sources

The Genome Wide Association Study (GWAS) data for AP in this research were obtained from the Finnish database (https://www.finngen.fi/en), which includes 6787 AP cases and 361,641 control participants. The GWAS data for the 46 antibody immune response factors were derived from a published study,^[24]^ comprising a total of 8735 samples, which include 46 antibody immune response phenotypes defined using data from 13 pathogens. The GWAS data for the 91 inflammatory cytokines were from a published study involving 11 cohorts, which include 14,824 participants.^[25]^ All participants included in our study were of European ancestry, effectively avoiding bias caused by racial differences and providing reliable conditions for our MR study. Additionally, the exposure samples in our study were derived from different GWAS datasets, ensuring that each sample is independent, which greatly reduces the possibility of sample overlap.

### 2.3. Selection of genetic IVs

We performed association analysis to select single-nucleotide polymorphisms (SNPs) closely related to exposure factors as IVs. When antibody immune response factors were considered as exposures, we set a genome-wide significance threshold of *P* < 1.0 × 10^−5^. When AP was considered as the exposure, we initially set the genome-wide significance threshold at *P* < 1.0 × 10^−6^, but due to the limited number of IVs obtained, the significance threshold was adjusted to *P* < 1.0 × 10^−5^. When 91 inflammatory cytokines were considered as exposures, we set the selection criteria for IVs to *P* < 1.0 × 10^−5^. These criteria ensured that the selected IVs were closely related to the exposure factors. To avoid genetic variation bias caused by linkage disequilibrium, we set the LD analysis criteria to *R*^2^ < 0.001 and a genetic distance of 10,000 kb. Additionally, to minimize and eliminate other confounding factors and weak instrument bias (*F*-statistic < 10), we selected SNPs with an *F*-statistic > 10 as IVs. The characteristics of the SNPs included in this research are listed in Tables S1, S2, and S3 (Supplemental Digital Content, https://links.lww.com/MD/Q348).

### 2.4. Two-sample MR analysis and sensitivity analysis

In the bidirectional two-sample MR analysis, we first used the inverse-variance weighted (IVW) method^[26,27]^ – a widely recognized, reliable, and effective method – to evaluate the causality between antibody immune response factors and AP. Furthermore, we employed the MR-Egger regression method,^[28,29]^ the weighted median method,^[30]^ the simple mode method, and the weighted mode method^[31]^ to perform the bidirectional two-sample MR analysis.

To ensure the reliability, robustness, and validity of the analysis results, we also utilized several sensitivity analysis methods, including Cochran *Q* test,^[27]^ MR-Egger intercept test,^[28]^ and MR-PRESSO method.^[32]^ Cochran *Q* test was used to evaluate heterogeneity in the results. If *P* < .05, heterogeneity was present; if *P* > .05, no heterogeneity was observed. If the instrumental variable affects the outcome through pathways other than the exposure factor, it indicates pleiotropy of the instrumental variable, violating the assumptions of independence and exclusivity.

We first utilized the MR-Egger intercept test to check pleiotropy. If *P* < .05, it indicates the presence of pleiotropy among the SNPs. In such cases, the MR-PRESSO method was applied to identify outliers, then reanalyzed the data after excluding the outliers to adjust for horizontal pleiotropy. We also conducted a leave-one-out sensitivity analysis to assess whether any individual SNPs had a significant influence on the causal relationship between exposure and outcome. The leave-one-out method^[33]^ involves sequentially removing each SNP to determine if a SNP has a large impact on the outcome, thereby evaluating the stability of the results. All data analyses were conducted using R Studio software version 4.3.3 with the “TwoSampleMR” and “MendelianRandomization” packages.

### 2.5. Mediation analysis of circulating inflammatory cytokines

Based on the results of the bidirectional two-sample MR analysis, we selected antibody immune response factors with positive results in the forward MR analysis and negative results in the reverse MR analysis for mediation analysis, to explore the mediating role of 91 inflammatory cytokines in their causal relationship. First, we calculated Beta1, representing the MR analysis with a specific antibody immune response factor as the exposure and inflammatory cytokines as the outcome. Then, we performed another MR analysis using the inflammatory cytokines as the exposure and AP as the outcome to calculate Beta2. The mediation effect was then calculated as Beta1 × Beta2.^[34]^ The detailed process of the mediation analysis is shown in Figure 1B.

## 3. Results

### 3.1. Results of genetic instrument selection

For the forward MR analysis, we screened and filtered the SNPs of 46 antibody immune response factors based on the criteria mentioned in the methods section. The results are presented in Table S1 (Supplemental Digital Content, https://links.lww.com/MD/Q348). The number of SNPs for each antibody immune response factor ranged from 5 to 59, with the *F*-statistic values between 20.776 and 343.063, indicating that all selected IVs were strong IVs. All selected SNPs were strongly associated with the antibody-mediated immune responses (*P* < 1.0 × 10^−5^).

In the reverse MR analysis, we considered AP as the exposure factor. Based on the screening criteria described in the methods section, 39 SNPs were selected as IVs, with the *F*-statistic values ranging from 19.55 to 73.82, demonstrating that all obtained IVs were strong IVs. All selected SNPs were strongly associated with AP (*P* < 1.0 × 10^−5^). The detailed results are shown in Table S2 (Supplemental Digital Content, https://links.lww.com/MD/Q348).

During the mediation analysis, with 91 inflammatory cytokines as the exposure factors, we screened and filtered their SNPs according to the criteria outlined in the methods section. The results are presented in Table S3 (Supplemental Digital Content, https://links.lww.com/MD/Q348). The *F*-statistic values for all SNPs ranged from 19.510 to 3549.334, indicating that all selected IVs were strong IVs. All selected IVs were significantly associated with the exposure factors (*P* < 1.0 × 10^−5^).

In summary, all selected IVs were significantly associated and strong, effectively avoiding bias caused by other confounding factors and weak IVs.

### 3.2. The impact of antibody immune response factors on AP

We analyzed the causal relationship between antibody-mediated immune responses and AP using the SNPs of 46 antibody immune response factors. Table S4 (Supplemental Digital Content, https://links.lww.com/MD/Q348) presents the results from 5 different analysis approaches: IVW, MR-Egger, weighted mode, weighted median, and simple mode.

**Figure 2.**
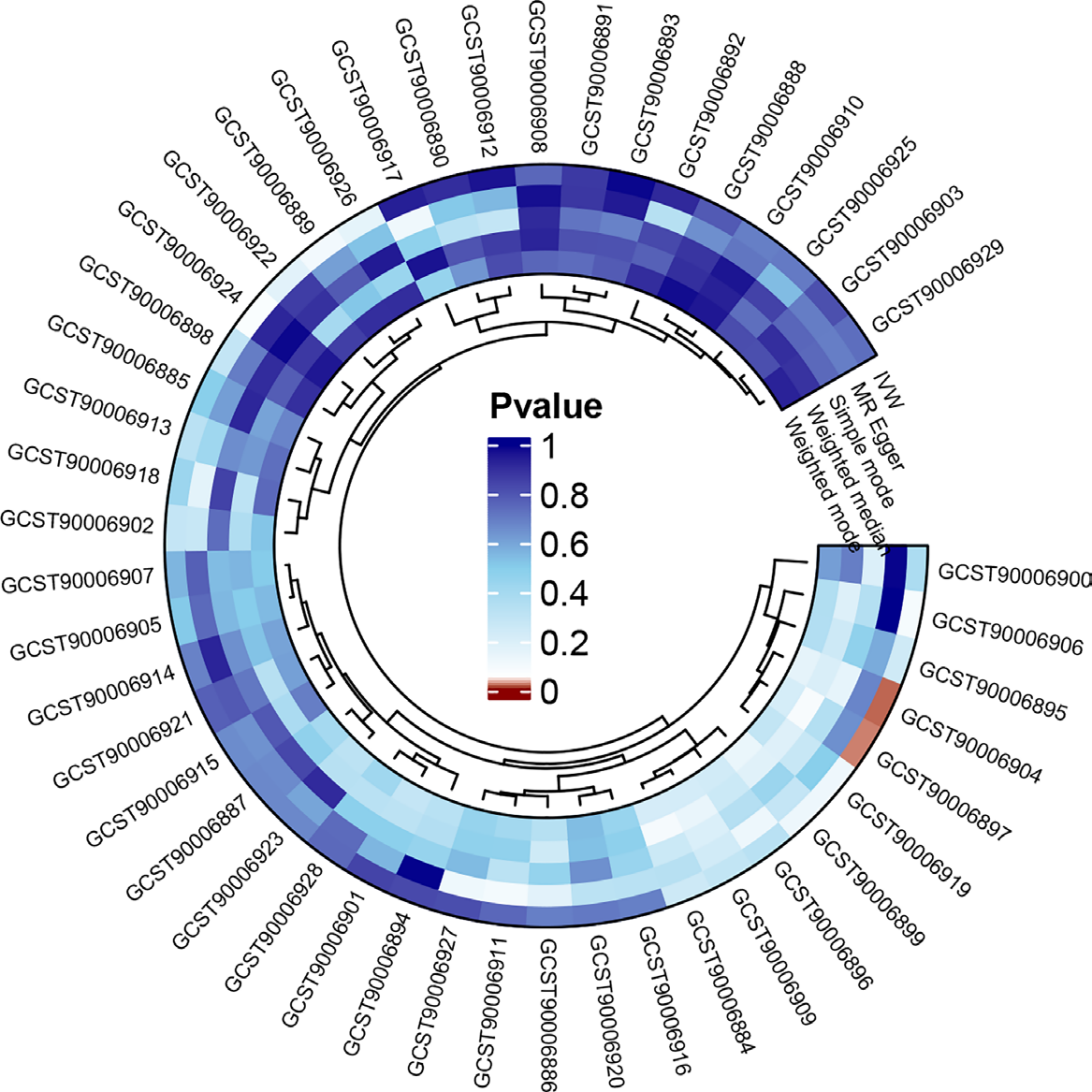
Positive loop diagram of the causality between 46 antibody-mediated immune responses and AP. AP = acute pancreatitis.

**Figure 3.**
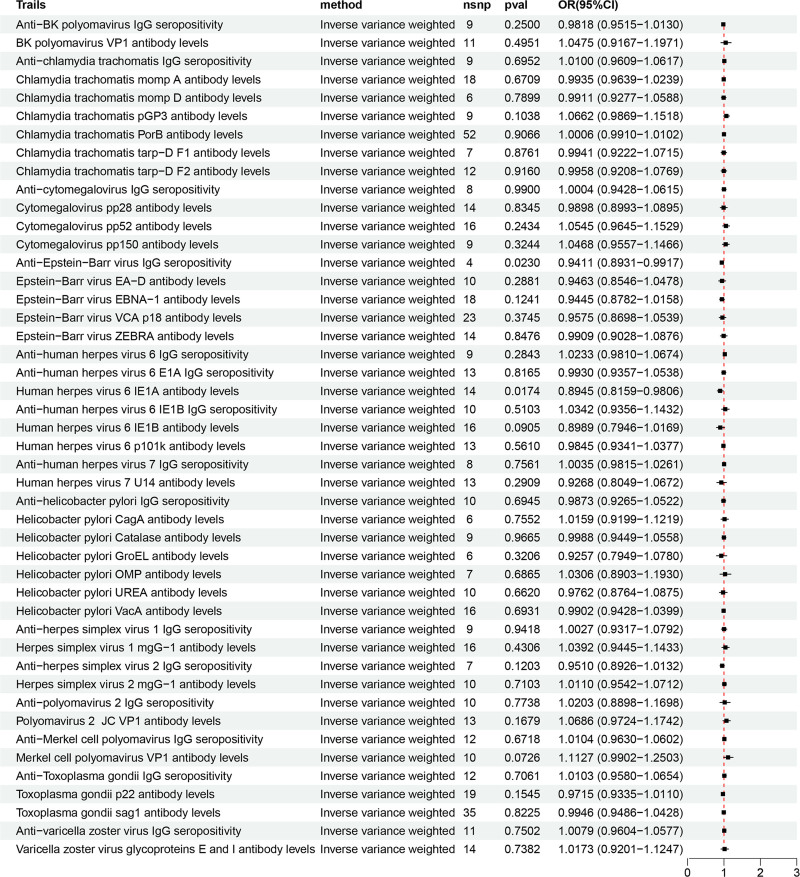
Forest plot of the causality between 46 antibody-mediated immune responses and acute pancreatitis in the results of forward MR analysis. MR = Mendelian randomization.

The IVW results supported a negative causal relationship between genetic susceptibility to two antibody immune response factors and the risk of AP. The causal relationship between the remaining 44 antibody immune response factors and AP risk was not statistically significant (Fig. 2). Specifically, anti-EBV IgG seropositivity [OR = 0.941; 95% CI, 0.893–0.992; *P* = .023] and human herpes virus (HHV)-6 IE1A antibody levels [OR = 0.894; 95% CI, 0.816–0.981; *P* = .017] were found to be the protective factors that could reduce the risk of AP (Fig. 3).

The results of Cochran *Q* test indicated no heterogeneity, while the MR-Egger intercept and MR-PRESSO tests indicated no horizontal pleiotropy. These findings suggest that the causal relationships between anti-EBV IgG seropositivity and HHV-6 IE1A antibody levels and AP are robust.

### 3.3. Impact of AP on antibody immune response factors

We conducted a reverse MR analysis using AP as the exposure and antibody immune response factors as the outcome to investigate whether genetic predisposition to AP influences antibody-mediated immune responses. The results from 5 different analysis methods are shown in Table S6 (Supplemental Digital Content, https://links.lww.com/MD/Q348).

**Figure 4.**
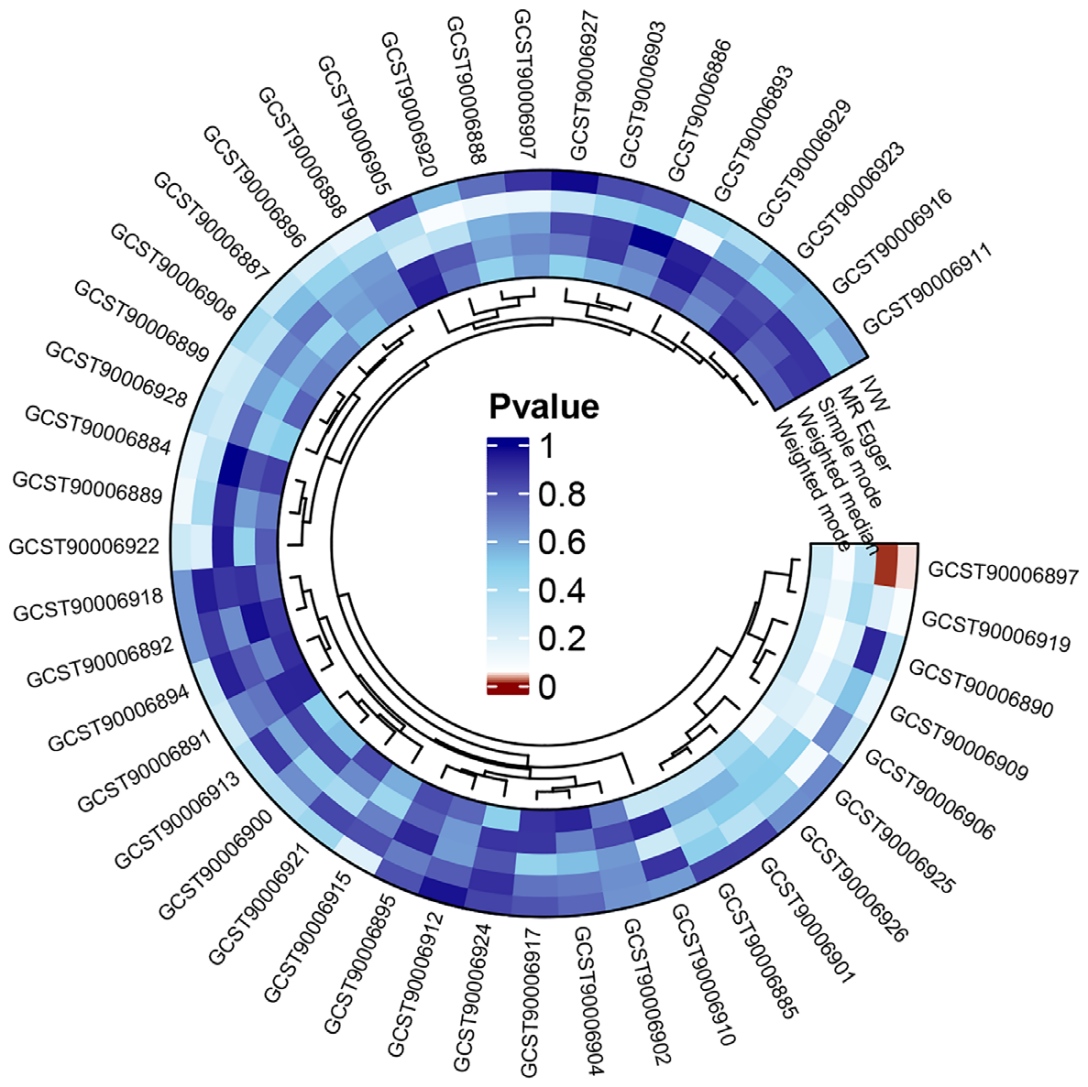
Reverse circular graph of the causality between AP and 46 antibody-mediated immune responses. AP = acute pancreatitis.

**Figure 5.**
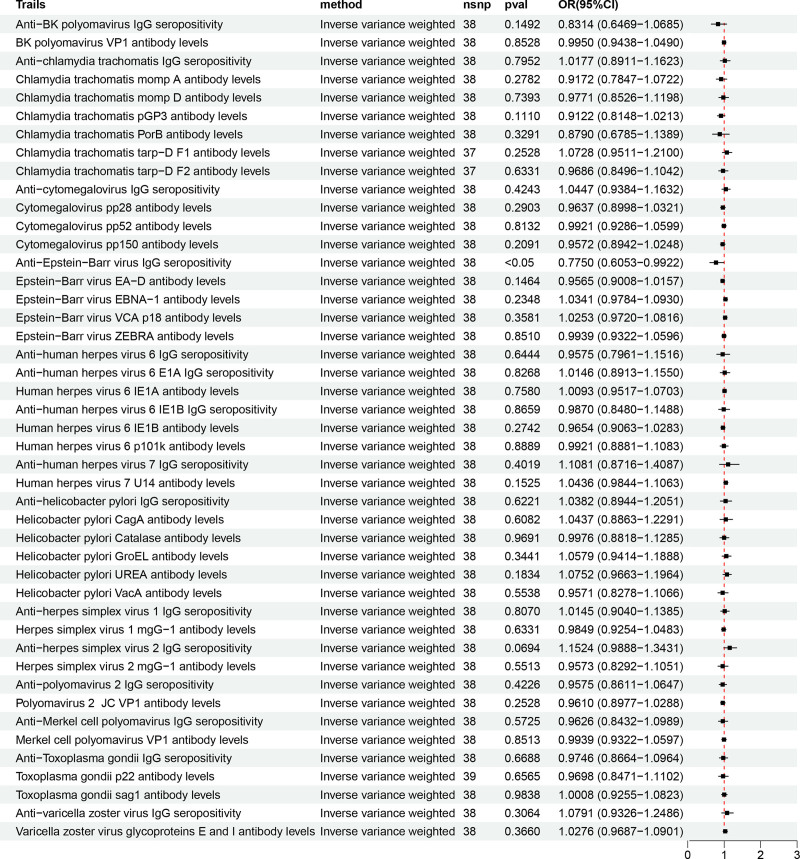
Forest plot of the causality between 46 antibody-mediated immune responses and acute pancreatitis in the results of reverse MR analysis.

We found that the genetic susceptibility of AP was significantly associated with only one antibody immune response factor, and not significantly related to the other 45 antibody immune response factors (Fig. 4). The preliminary IVW results revealed a negative causal relationship between genetic susceptibility to AP and anti-EBV IgG seropositivity [OR = 0.775; 95% CI, 0.605–0.992; *P* = .043] (Fig. 5). The MR-Egger results also supported this conclusion (OR = 0.531; 95% CI, 0.346–0.814; *P* = .006; Table S6, Supplemental Digital Content, https://links.lww.com/MD/Q348). The Cochran *Q* test results showed no heterogeneity in the reverse positive results. Although the MR-Egger intercept test detected horizontal pleiotropy in this causal relationship (*P* < .05), after excluding outliers and correcting the statistical analysis using the MR-PRESSO global test, the horizontal pleiotropy disappeared (*P* > .05), confirming the robustness of our MR analysis results.

### 3.4. Sensitivity analysis

**Figure 6.**
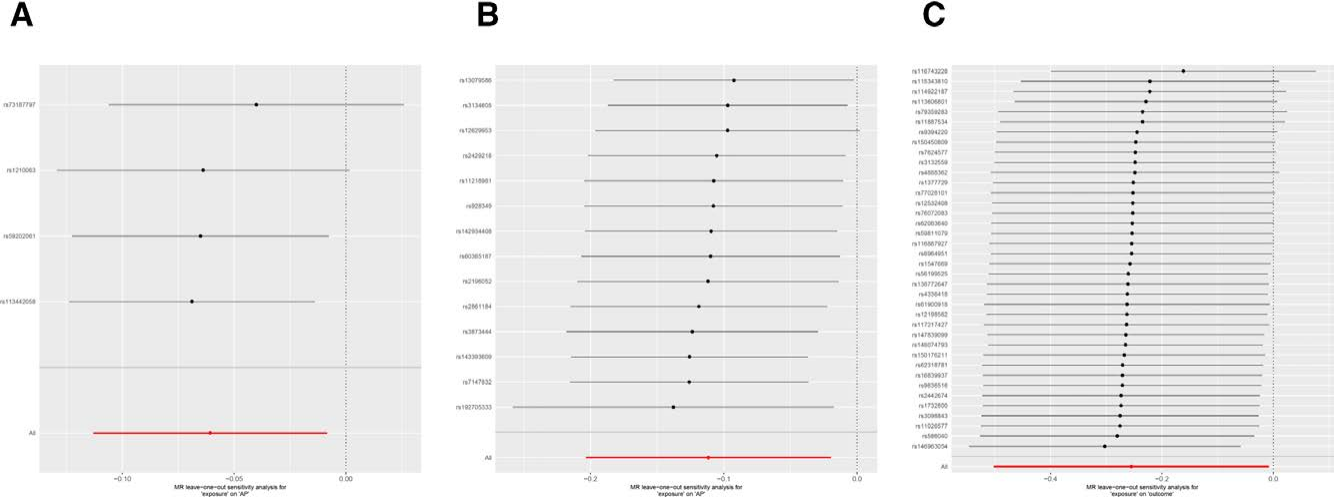
Leave-one-out sensitivity analysis plots. (A) Forward leave-one-out sensitivity analysis plot for anti-EBV IgG seropositivity; (B) forward leave-one-out sensitivity analysis plot for HHV-6 IE1A antibody levels; (C) reverse leave-one-out sensitivity analysis plot for anti-EBV IgG seropositivity. Anti-EBV = anti-Epstein-Barr virus, HHV = human herpesvirus.

We employed several different sensitivity analysis methods to determine whether there was any heterogeneity or horizontal pleiotropy in the results. The sensitivity analysis results for the MR analysis are presented in Tables S5 and S7 (Supplemental Digital Content, https://links.lww.com/MD/Q348). Cochran *Q* test results showed that a few statistics exhibited heterogeneity; however, after applying the random effects model, the estimates did not change significantly. The MR-Egger intercept test indicated that a few statistics had horizontal pleiotropy (*P* < .05). Still, after excluding outliers and correcting the statistical analysis using the MR-PRESSO global test, all estimates indicated no horizontal pleiotropy. These results indicate that our MR analysis results are robust. Moreover, the leave-one-out sensitivity analysis indicated that there was no causality between any single SNP and the positive results (Fig. 6).

### 3.5. Mediation analysis

The forward MR results indicated that HHV-6 IE1A antibody levels significantly reduce the risk of AP, and there is no reverse causal relationship. However, whether this causal relationship was mediated by inflammatory cytokines remains unclear. We employed a two-step MR analysis method to explore whether any of the 91 inflammatory cytokines serve as mediators in this causal relationship.

First, we performed an MR analysis between HHV-6 IE1A antibody levels and the 91 circulating inflammatory cytokines. The statistical results of the different MR methods are shown in Table S8 (Supplemental Digital Content, https://links.lww.com/MD/Q348). The initial IVW results showed a significant positive correlation between HHV-6 IE1A antibody levels and Caspase 8 levels [OR = 1.078; 95% CI, 1.007–1.155; *P* = .032], Leukemia inhibitory factor receptor levels [OR = 1.081; 95% CI, 1.005–1.162; *P* = .037], and Stem cell factor levels [OR = 1.087; 95% CI, 1.015–1.164; *P* = .017]. However, these 3 circulating inflammatory cytokines were not influenced by genetic susceptibility to AP (Table S9, Supplemental Digital Content, https://links.lww.com/MD/Q348). The results of the sensitivity analysis also showed no significant heterogeneity and horizontal pleiotropy (Table S8, Supplemental Digital Content, https://links.lww.com/MD/Q348).

Also, we conducted an MR analysis between the 91 circulating inflammatory cytokines and AP. The statistical results of the different MR approaches are presented in Table S9 (Supplemental Digital Content, https://links.lww.com/MD/Q348). The IVW results showed a significant positive correlation between the risk of AP and Interleukin-15 receptor subunit alpha levels [OR = 1.128; 95% CI, 1.056–1.204; *P* = .000], Interleukin-1-alpha levels [OR = 1.115; 95% CI, 1.003–1.239; *P* = .044], Interleukin-2 receptor subunit beta levels [OR = 1.116; 95% CI, 1.005–1.240; *P* = .040], Monocyte chemoattractant protein-4 levels [OR = 1.117; 95% CI, 1.033–1.208; *P* = .006], and Tumor necrosis factor receptor superfamily member 9 levels [OR = 1.157; 95% CI, 1.051–1.273; *P* = .003]. However, no significant causality was found between these 5 inflammatory cytokines and HHV-6 IE1A antibody levels (Table S8, Supplemental Digital Content, https://links.lww.com/MD/Q348). The sensitivity analysis results also confirmed no significant heterogeneity or horizontal pleiotropy (Table S9, Supplemental Digital Content, https://links.lww.com/MD/Q348).

Overall, there is no evidence to indicate that any of the 91 inflammatory cytokines mediate the causality between HHV-6 IE1A antibody levels and reduced risk of AP.

## 4. Discussion

As far as we know, our study is the first to investigate the causality between 46 antibody immune response factors and AP using the largest and most comprehensive GWAS dataset, and it is also the first one to perform a mediation analysis using the largest number of inflammatory cytokines. The findings suggest that genetic susceptibility to anti-EBV IgG and HHV-6 IE1A antibodies is negatively associated with AP, and there is a reverse causality between anti-EBV IgG and AP. The sensitivity analysis results indicated that these causal relationships are robust. Furthermore, the mediation analysis suggests that inflammatory cytokines may not mediate the causal relationship between HHV-6 IE1A antibody levels and AP. Overall, the current study’s results provide some reference value for antibody-mediated immune responses in the prevention and treatment of AP.

Epstein-Barr virus (EBV) is a widespread lymphotropic herpesvirus that infects humans and is transmitted through close contact between susceptible individuals and asymptomatic EBV carriers. It is a type of IV herpesvirus. EBV has latent and transformative properties.^[35]^ Globally, more than 90% of adults have been infected with EBV at some point. EBV infection can lead to various cancers, such as nasopharyngeal carcinoma and gastric cancer.^[36]^

B cells are the primary host cells for EBV. After infection, EBV remains latent in the body’s memory B cells for life, typically without causing any symptoms.^[35]^ Following EBV infection, the body produces EBV IgG, which serves as an endogenous antigen, and anti-EBV IgG, mediating the antibody immune response. Anti-EBV IgG seropositivity not only reflects previous infections, but also essentially embodies the immune control ability of the body to maintain latent EBV infection – this ability is regulated by genetically determined immune response characteristics (such as B-cell memory formation efficiency, antibody affinity maturity) – but it cannot determine whether the current infection is in the active stage.

Clinical studies reported that serum tests of patients with EBV-associated AP showed seropositivity for EBV IgG.^[37,38]^ In fact, research indicated that 82.4% of patients with EBV infection might develop AP.^[39,40]^ Other studies suggested that serum markers indicated that EBV infection causes AP, which is an atypical manifestation of EBV infection.^[41,42]^ Clinical reports also suggested that EBV might cause immune changes in the liver or induce the release of inflammatory mediators and cytokines, leading to pancreatitis.^[43]^ However, our MR analysis yielded opposite results, indicating that anti-EBV IgG seropositivity significantly reduces the risk of AP, and sensitivity analysis proved the robustness of the MR findings.

What could be the reason for this contradictory conclusion? We believe that previous studies were mainly based on case reports or clinical observational studies, which were vulnerable to confounding factors. However, our study applied MR methods which use Mendel genetic laws as IVs to effectively avoid the impact of common confounding factors. Our MR study investigated exposure to anti-EBV IgG seropositivity that is genetically determined and reflects an individual’s ability to maintain long-term latent EBV infection. This is a key difference from the “EBV-associated AP” reported in previous observational studies: the latter typically refers to evidence of active EBV infection, such as viral reactivation, detected at the onset of AP. We speculate that stable, genetically determined immune control against EBV, as indicated by lifelong IgG levels, may be protective against EBV reactivation and associated inflammation that spreads to the pancreas. EBV reactivation (active infection) may increase the risk of AP through the aforementioned mechanisms such as immune dysregulation and cytokine storm. Thus, the seemingly contradictory results may in fact reflect the different effects of different stages of EBV infection – stable latency versus reactivation of activity – on AP risk. Therefore, the MR conclusions are more reliable. Currently, there is no direct evidence to confirm a reverse causality between anti-EBV IgG seropositivity and AP risk. Larger-scale clinical studies are needed in the future to further explore the causality between EBV-related antibodies and AP risk.

HHV-6 belongs to the Betaherpesvirinae subfamily of the Herpesviridae family and was a new virus obtained in 1986 from the lymphocytes of patients with lymphoproliferative disorders (LPD) and acquired immunodeficiency syndrome (AIDS). HHV-6 variants are classified into HHV-6A and HHV-6B, with HHV-6A being more common in immunocompromised hosts.^[44]^ Clinical studies found that HHV-6A can cause pancreatitis in immunosuppressed individuals.^[45]^ Studies also showed that primary HHV-6A infection can lead to hepatitis in immunosuppressed individuals, and all types of hepatitis may be complicated by AP.^[46,47]^ Clinical evidence indicated that immune responses mediated by HHV-6 antigen-antibody interactions play a crucial role in controlling primary and latent infections, preventing or treating HHV-6-related complications such as fulminant hepatitis, which indirectly reduces the risk of AP.^[48,49]^ Clinical evidence also indicated that the reactivation of HHV-6 is related to cytokines such as IL-22 and TNF-α, and these cytokines have protective effects in AP.^[50–52]^ The current study’s MR results showed that HHV-6 IE1A antibody levels can reduce the risk of AP, and there is no reverse causal relationship. This evidence strongly supports the reliability of the MR results, and the sensitivity analysis further confirms the robustness of our results.

Moreover, our mediation analysis indicated that inflammatory cytokines do not mediate the causal relationship between HHV-6 IE1A antibody levels and reduced AP risk. This causal relationship may be mediated by other factors, such as cellular immunity and gut microbiota. One study found that HHV-6-specific T lymphocytes were apparently elevated in patients who reactivate the virus.^[53]^ A report suggested that T-cell immunotherapy can prevent HHV-6 infection in immunocompromised patients and reduce the probability of viral reactivation.^[54]^ Another clinical study indicated that nicotine could improve experimental SAP by enhancing the immune regulation of CD4 + and CD25 + regulatory T cells.^[55]^ Furthermore, a study indicated that the immunoregulation of specific immune cell populations, including CD8 + T cells and CD4 + T cells, could be potential therapeutic targets for AP, which requires further investigation.^[56]^

Previous research suggested that HHV-6 primarily infects NK cells, monocyte-macrophages, CD4 + T cells, and CD8 + T cells, and gastrointestinal diseases are related to the reactivation of HHV-6, but the causality remains unclear and needs further study.^[57]^ Some other studies’ MR analysis reports showed a causality between the gut microbiota and gastrointestinal diseases such as AP.^[58,59]^ A study suggested that maintaining the balance of the gut microbiome may reduce the risk of AP.^[60]^ Additionally, research suggested that metabolites of the gut microbiota such as short-chain fatty acids, can suppress inflammatory responses, thereby preventing and improving pancreatic injury.^[61]^ This evidence suggested that the gut microbiota and cellular immunity may mediate the relationship between HHV-6 IE1A antibody levels and AP risk. Future large-scale clinical research is needed to further explore the mediating roles of cellular immunity and gut microbiota in the causal relationship between HHV-6 IE1A antibody levels and AP risk, to fully elucidate the exact causal relationship between them. The results will provide valuable guidance for the clinical prevention and treatment of AP, potentially reducing its incidence and mortality.

The significance and advantages of this study include: (1) The MR analysis used data from large-scale GWAS databases that are publicly available. By examining the bidirectional causality between antibody-mediated immune responses and AP from a genetic epidemiology perspective, we proved that both anti-EBV IgG seropositivity and HHV-6 IE1A antibody levels significantly reduce the risk of AP, with AP showing a significant negative correlation with anti-EBV IgG seropositivity. This provides valuable insights into the clinical prevention and treatment of AP in the future. (2) A two-step MR analysis was performed to evaluate whether 91 circulating inflammatory cytokines mediate and influence the causality between antibody-mediated immune responses and AP. Our results indicated that inflammatory cytokines do not act as mediators in the causal relationship between the 2. (3) This MR study employed several analytical methods to investigate the correlation between antibody-mediated immune responses and AP and employed a two-step method to assess the intermediary role of the 91 circulating inflammatory cytokines. The sensitivity analysis results confirmed that the MR findings are reliable. Compared to traditional epidemiological research, the major advantage of our MR study is its ability to avoid various confounding factors and reverse causality, saving both time and effort.

In addition, this study has the following implications for future research and clinical practice: (1) The forward MR analysis result indicated that genetic susceptibility to anti-EBV IgG seropositivity and HHV-6 IE1A antibody levels are inversely related to AP risk. The reverse MR analysis showed that genetic susceptibility to AP is also inversely related to the risk of anti-EBV IgG seropositivity, suggesting a bidirectional protective causal relationship between antibody-mediated immune responses and AP. However, currently few studies directly confirm these causal relationships. Future research could involve experimental studies to directly verify these potential causal relationships and to provide valuable guidance for the precise clinical prevention and treatment of AP. (2) Previous genetic epidemiological studies suggested a bidirectional causality between AP and antibody-mediated immune responses. However, the specific genetic and biological mechanisms remain unclear. Future research could further explore these mechanisms to better reduce the incidence and mortality of AP patients. (3) The MR analysis results indicated that anti-EBV IgG seropositivity significantly reduces the risk of AP, but no existing literature confirms this finding. Future studies could further investigate to clarify the potential causal relationship between anti-EBV IgG seropositivity and AP. (4) This study’s data were primarily derived from European populations. Future research could consider including data from other regions or ethnicities to improve the global applicability and generalizability of the results. (5) Further studies could also explore whether there is a bidirectional causality between HHV-6 IE1A antibody levels and AP, and whether the genetic susceptibility of this antibody immune response serves as a protective factor against AP.

The limitations of this study include: (1) All GWAS data used in this study are derived from European populations, and it remains to be determined whether our findings are applicable to other regions or ethnicities; (2) The study primarily focused on genetic factors and did not fully consider the effects of confounding factors, such as ethnicity, socioeconomic status, and environment, on the causality between antibody-mediated immune responses and AP. (3) The GWAS database has certain limitations, including the inability to exclude the impact of factors such as sex and age on the study results. Besides, the GWAS data did not include stratified analyses based on the severity of AP, nor do they contain data on SAP. In the future, larger-scale, prospective designed studies specifically targeting SAP for mediating analysis will be conducted.

## 5. Conclusion

The current study’s findings suggest that HHV-6 IE1A antibody levels are a protective factor against AP, and there is a bidirectional causal relationship between AP and anti-EBV IgG seropositivity. Moreover, the mediation analysis results indicate that the 91 circulating inflammatory cytokines do not act as mediators between the 46 antibody-mediated immune responses and AP. These findings provide new insights into the pathogenesis and pathological mechanisms of AP, making an important and positive contribution to its prevention and treatment.

## Acknowledgments

The authors would like to convey their heartfelt appreciation to the researchers who generously shared the GWAS data we utilized.

## Author contributions

**Conceptualization:** Sheng Xie.

**Data curation:** Minjian Xie, Liqun Li, Lijian Liu.

**Formal analysis:** Minjian Xie, Liqun Li, Lijian Liu.

**Project administration:** Sheng Xie.

**Software:** Chengning Yang.

**Visualization:** Xiaoyun Lei.

**Writing – original draft:** Minjian Xie, Liqun Li, Lijian Liu.

**Writing – review & editing:** Jianfeng Li.

## Supplementary Material


